# Handheld Nonthermal Plasma Augmentation of Glass–Ceramic Spray Deposition on Zirconia Surface Characterization and MG-63/HGF-1 Cell Behavior: An In Vitro Study

**DOI:** 10.3390/jfb16110421

**Published:** 2025-11-11

**Authors:** Sheng-Han Wu, Szu-Yu Lai, I-Ta Lee, Yuichi Mine, Huei-Yu Huang, Tzu-Yu Peng

**Affiliations:** 1 School of Dentistry, College of Oral Medicine, Taipei Medical University, Taipei 11031, Taiwanitlee0128@tmu.edu.tw (I.-T.L.); 2 Department of Medical Systems Engineering, Graduate School of Biomedical and Health Sciences, Hiroshima University, Hiroshima City 734-8553, Japan; 3 Department of Oral Medicine, Taipei Medical University-Shuang Ho Hospital, New Taipei City 23561, Taiwan

**Keywords:** zirconia, glass–ceramic spray deposition, handheld nonthermal plasma, wettability, surface roughness, cell attachment, bone metabolic activity, gingival fibroblasts, biocompatibility, alkaline phosphatase

## Abstract

Zirconia is widely used for customized implant abutments owing to its esthetics, strength, and biocompatibility; however, the optimal surface modification for soft-tissue sealing and bone metabolic remains uncertain. This study evaluated how glass–ceramic spray deposition (GCSD), with or without handheld nonthermal plasma (HNP), alters zirconia surface physiochemistry and cellular responses. Field-emission scanning electron microscopy/energy-dispersive X-ray spectroscopy, surface roughness (Ra), wettability, and surface free energy (SFE) were measured. Human osteoblast-like cells (MG-63) and human gingival fibroblasts (HGF-1) were used to assess attachment and spreading, metabolic activity, cytotoxicity, and inflammatory response (tumor necrosis factor-α, TNF-α) (α = 0.05). GCSD produced an interlaced rod- and needle-like glass–ceramic layer, significantly increasing Ra and hydrophilicity. HNP further reduced surface contaminants, increased SFE, and enhanced wettability. The combination of GCSD and HNP yielded the greatest attachment and spreading for both cell types, without increases in cytotoxicity or TNF-α. GCSD with HNP creates a hydrophilic, micro-textured, chemically activated zirconia surface that maintains biocompatibility while promoting early attachment and bone metabolic activity, supporting its application for zirconia implant abutments.

## 1. Introduction

Dental implants have become a cornerstone of modern oral rehabilitation, restoring function and esthetics with high long-term survival when biological and prosthetic principles are respected. Within this context, the choice and surface state of materials at the transmucosal interface are critical, because they influence soft-tissue sealing, biofilm control, and ultimately peri-implant health over time [1,2]. Zirconia is a fully crystalline ceramic widely used in dentistry [1,2]. At room temperature, it naturally adopts a monoclinic phase (m-ZrO_2_), whereas the tetragonal phase (t-ZrO_2_) exhibits superior physical properties [3,4]. To stabilize t-ZrO_2_ at room temperature, metal-oxide dopants (e.g., Y_2_O_3_, CaO, Al_2_O_3_, CeO_2_, MgO) are incorporated to modify the crystal lattice [1,5]. The excellent mechanical performance of zirconia arises from transformation toughening in which under applied stress, t-ZrO_2_ locally transforms to m-ZrO_2_, producing approx. 3–5% volumetric expansion that generates compressive stresses at crack tips and suppresses crack propagation [6,7]. The effectiveness of this mechanism depends on grain size and phase composition; grains should be near the critical size (approx. 1 μm) to retain tetragonal stability, whereas very small grains (<0.2 μm) or excessive cubic content (c-ZrO_2_) diminish transformation toughening and lower strength [8]. Adjusting stabilizer content also tunes zirconia translucency to meet esthetic demands [9,10]. In addition, zirconia shows high biocompatibility, a smooth, plaque-resistant surface, and superior corrosion and chemical stability compared with titanium, without the risk of metal hypersensitivity [11,12]. These attributes explain the increasing use of zirconia at the peri-implant transmucosal zone, either as a monolithic abutment segment or as a zirconia emergence profile on a Ti-base.

The implant abutment primarily interfaces with peri-implant soft tissues and the oral environment; its internal (apical) connection engages the implant but does not contact bone in dental practice. Thus, beyond mechanical integrity, the transmucosal abutment surface should support a stable epithelial/gingival connective-tissue seal that limits bacterial ingress at the implant–abutment microgap and reduces the risk of peri-implant mucositis [13,14,15]. Titanium and zirconia are the principal abutment materials; however, zirconia additionally offers superior esthetics, chemical stability, and freedom from metal allergy, making it increasingly popular [16,17]. With the growth of digital dentistry, customized zirconia abutments have become mainstream [18]. While polished zirconia is widely regarded as a standard for hygiene, there is ongoing interest in whether carefully engineered surface chemistry/wettability, without macro-roughening, could further enhance early soft-tissue attachment and biological sealing.

Glass–ceramic spray deposition (GCSD) is an emerging surface treatment for zirconia [19]. In GCSD, a nanosilica–lithium precursor is uniformly spray-deposited onto zirconia and heat-treated in a dental furnace to form a dense glass–ceramic layer [20,21]. Subsequent hydrofluoric (HF) etching exposes the matrix, producing a micro-/nano-textured and cleaned surface [22,23]. By combining the biofriendly attributes of lithium disilicate-based glass–ceramics with the mechanical advantages of zirconia, GCSD increases surface chemical reactivity and micromechanical interlocking, thereby improving bonding to resin cements and overcoming the limited roughness and inert surface chemistry of zirconia [19,20,21,22,23]. Importantly, for the transmucosal region, GCSD can be tuned to increase surface energy and protein adsorption while maintaining surface roughness within clinically acceptable “smooth” ranges, avoiding the plaque-retentive effects of macro-roughness [24,25]. From a biomechanical perspective, the elastic modulus of glass–ceramics is between that of zirconia and bone, which can reduce the direct transmission of occlusal forces, have a shock-absorbing effect, and promote more uniform load transfer [26,27,28]. Practically, GCSD is efficient, low-cost, and amenable to standardized workflows without specialized equipment, supporting clinical translation. In this study, we consider GCSD as a thin, functional interlayer (distinct from cosmetic glazing) aimed at modulating interfacial chemistry rather than altering internal connection geometry or fit.

Plasma is another surface-activation approach of growing interest. Nonthermal plasma introduces reactive species that remove organic contaminants without altering surface topography, while generating surface oxides and hydroxyl –OH groups that increase SFE and wettability [15,29]. These changes can attenuate inflammatory responses and promote enhanced cell spreading, a more robust actin cytoskeleton, and stronger Ca^2+^ signaling, collectively supporting bone metabolic activity and soft-tissue integration at the transmucosal interface [15,30]. Portable, handheld nonthermal plasma HNP devices enable chairside application, offering short treatment times without high temperatures or vacuum, and have demonstrated effective surface modification compatible with existing protocols [29,31,32]. Because the plasma is dry and residue-free, it suits abutments with complex geometries and heat-sensitive components, improves initial protein adsorption profiles linked to favorable cell attachment, and may reduce early bacterial adhesion [15,29,31,32]. Clinically, HNP is versatile for pre-bond decontamination during cementation and for periodic maintenance of transmucosal surfaces at recall, integrating seamlessly into existing chairside workflows [29,31,32].

Given a durable soft-tissue seal, we explored whether combining GCSD (to tailor surface chemistry/energy) with HNP (for decontamination and hydrophilicity) could enhance early epithelial/gingival fibroblast responses on zirconia abutment-like surfaces, without increasing plaque-retentive roughness or affecting component fit. Evidence remains limited regarding how such treatments jointly impact zirconia physicochemical features and soft-tissue-relevant cell behavior in vitro. Accordingly, the present study examines the individual and combined impacts of GCSD and HNP on zirconia surfaces and evaluates cellular outcomes in vitro using human osteoblast-like cells (MG-63) and human gingival fibroblasts (HGF-1) as complementary models. Herein, this in vitro study hypothesized that GCSD coupled with HNP would increase surface energy and cleanliness while keeping roughness within acceptable limits, thereby improving early cell attachment and selected functional readouts relevant to soft-tissue sealing.

## 2. Materials and Methods

### 2.1. Sample Preparation

Disk-shaped designs (diameter 10 mm; thickness 2.5 mm) were created in SolidWorks 2019 (Dassault Systèmes, Providence, RI, USA). Zirconia (Superfect Zir; Aidite Technology Co., Ltd., Qinhuangdao, China) and titanium alloy (Ti-6Al-4V; S-Tech Corp., Tainan, Taiwan) disks were milled using a dental CAD/CAM unit (Cameo 250i; Aidite Technology). All samples were sequentially ground with silicon-carbide papers (#320, #400, #600), then ultrasonically cleaned in isopropanol and distilled water for 10 min each (UC-4060HL; Shenzhen Jiayuanda Technology, Shenzheng, China).

### 2.2. Surface Treatments

**Figure 1:**
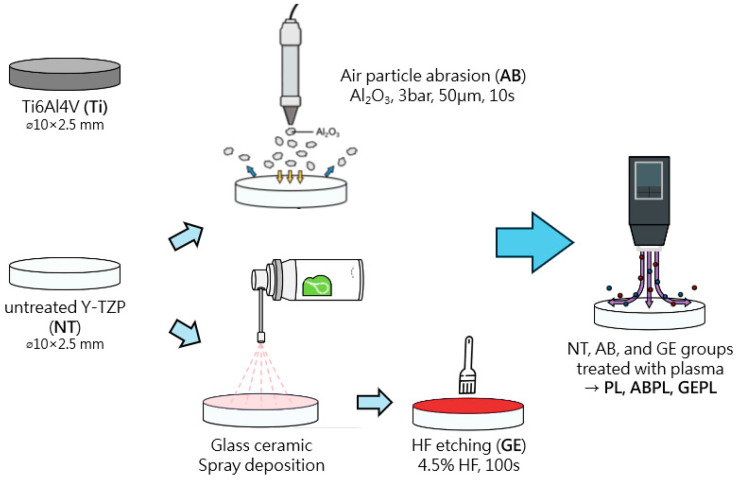
Experimental workflow, grouping, and abbreviation.

Samples were randomly allocated to seven groups (n = 10 per group) as shown in Figure 1.

(1) NT group: Ground only.
(2) PL group: HNP (PiezoBrush^®^ PZ3; relyon Plasma GmbH, Regensburg, Germany) at 18 W/50 kHz for 30 s.
(3) AB group: Airborne-particle abrasion with 50 µm Al_2_O_3_ (Cobra; Renfert GmbH, Hilzingen, Germany) at 3 bar, 10 mm standoff, 10 s.
(4) ABPL group: AB followed by HNP.
(5) GE group: GCSD using a glass–ceramic powder (Biomic LiSi connector; Aidite) sprayed uniformly at 10 mm standoff, then processed in a dental furnace (AUSTROMAT 220; DEKEMA Dental-Keramiköfen GmbH, Freilassing, Germany): 450 °C drying for 1 min; heating at 80 °C/min to 895 °C with a 1.5 min hold; controlled cooling at 10 °C/min to 25 °C; etched with 4.5% hydrofluoric (HF) acid (IPS Ceramic Etching Gel; Ivoclar Vivadent, Schaan, Liechtenstein) for 100 s.
(6) GEPL group: GCSD followed by HNP.
(7) Ti group (baseline): Ground titanium.

### 2.3. Surface Morphology and Elemental Analyses

Surface morphology and microstructure were examined using field-emission scanning electron microscopy (FE-SEM; JSM-7800F Prime, JEOL Ltd., Tokyo, Japan). Samples were mounted with carbon tape and sputter-coated with platinum using an auto fine coater (JEC-3000FC, JEOL Ltd.) at 10 mA for 25 s. Images were recorded at 3.0 kV at ×500 and ×2500 (n = 3). Elemental composition and distribution were characterized by energy-dispersive X-ray spectroscopy (EDS; integrated on the JSM-7800F Prime) (n = 3).

### 2.4. Surface Wettability and Roughness

Static contact angles (CAs) were measured with a contact angle analyzer (Phoenix Mini; Surface Electro Optics, Suwon-s, Republic of Korea) using the sessile-drop method with distilled water (polar) and diiodomethane (non-polar). Images were acquired 5 s after deposition (n = 10). Surface free energy (SFE) was calculated from the two-liquid CAs using the Owens–Wendt–Rabel–Kaelble method (Surfaceware; Surface Electro Optics) (n = 10).

Surface roughness was assessed with a surface roughness tester (SurfTest SJ-210, Mitutoyo Co., Ltd., Kawasaki City, Japan) following relevant ISO 1997 settings [33]. A diamond stylus traversed 6.4 mm at 0.5 mm/s perpendicular to the surface, and arithmetic mean roughness (Ra) was recorded (n = 10). Four locations per sample were averaged.

### 2.5. Cell Culture

Samples were autoclaved at 121 °C and 1.1 kg/cm^2^ for 30 min and dried at 55 °C. Unless otherwise noted, each assay used n = 5/group and was performed in triplicate.

MG-63 cells (Basel Convention Regional Centre, Hsinchu, Taiwan) were maintained in minimum essential medium (MEM; Invitrogen, Carlsbad, CA, USA) with 10% fetal bovine serum (FBS) and 1% penicillin–streptomycin (100 U/mL penicillin, 100 µg/mL streptomycin). HGF-1 cells (American Type Culture Collection, Manassas, VA, USA) were cultured in Dulbecco’s modified Eagle’s medium (DMEM; Invitrogen) with 10% FBS and 1% penicillin–streptomycin. Cells were incubated at 37 °C in 5% CO_2_ and fed every 2 days. At approx. 80% confluence, cells were rinsed with phosphate-buffered saline (PBS), detached with trypsin–ethylenediaminetetraacetic acid for 2 min at 37 °C, neutralized, centrifuged at 1200 rpm for 5 min at 4 °C, and resuspended for seeding.

### 2.6. Cell Attachment

Surface-treated samples were placed in 24-well plates. MG-63 cells were seeded at 5 × 10^4^ cells/mL and incubated for 8 h; HGF-1 cells were seeded at the same density and incubated for 24 h. Wells were aspirated, rinsed twice with PBS, fixed with 10% neutral-buffered formalin for 15 min at 25 °C, rinsed, and dehydrated through graded ethanol (35–99%; 15 min each). After drying, samples were mounted and examined by FE-SEM. The medical imaging software program (NIH ImageJ 1.54g; National Institutes of Health, Bethesda, MD, USA) was used for binary thresholding to quantify cell-covered area (%) as a proxy for attachment.

### 2.7. Cell Metabolic Activity

MG-63 and HGF-1 (5 × 10^4^ cells/mL) were cultured on disks for 1, 3, and 7 days. At each time point, 50 µL of PrestoBlue reagent (Invitrogen) was added per well and incubated for 1 h at 37 °C. Absorbance was read at 570 nm with a 600 nm reference (Spark 10 M; Tecan, Männedorf, Switzerland).

### 2.8. Cytotoxicity

Surface-treated samples were immersed in complete medium (5 mL) at 37 °C/5% CO_2_ for 1, 3, or 7 days to obtain extracts. MG-63 and HGF-1 at approx. 80% confluence was exposed to the corresponding extracts for 24 h. PrestoBlue (50 µL/well) was added for 1 h at 37 °C, and absorbance was recorded at 570/600 nm.

### 2.9. Inflammatory Response

MG-63 and HGF-1 were seeded on the surfaces (5 × 10^4^ cells/mL) and cultured for 7 days, with medium exchange every 2 days. Spent media were stored at −20 °C. TNF-α secretion was quantified by ELISA (Invitrogen) according to the manufacturer’s instructions. Absorbance was measured at 450 nm with a 570/600 nm reference.

### 2.10. Statistical Analysis

Normality and homogeneity of variance were assessed using the Shapiro–Wilk and Levene tests, respectively. When assumptions were met, parametric statistics were used. One-way analysis of variance compared wettability, SFE, roughness, viability, and cytotoxicity across groups, followed by Tukey’s honestly significant difference post hoc tests. Analyses were performed in GraphPad Prism 10 (GraphPad Software, San Diego, CA, USA). Statistical significance was set at α = 0.05.

## 3. Results

### 3.1. Surface Characteristics

**Figure 2:**
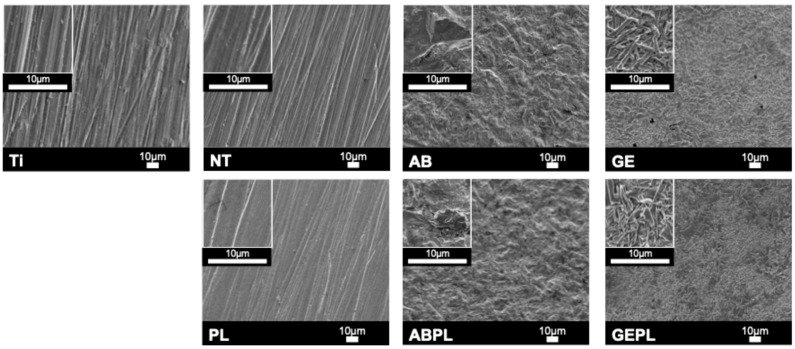
Surface micrographs. Main panels: SEM at 500×; inset (**upper-left**): 2500×.

Figure 2 shows FE-SEM microtopography for each group. Ti, NT, and PL surfaces appeared homogeneous and flat, with fine polishing striations. AB and ABPL exhibited irregular, undulating roughness with pronounced relief. After HF etching, GE and GEPL displayed a precipitated glassy phase where rod-like lithium metasilicate and needle-like lithium disilicate interlaced to form a dense, interlocking glass–ceramic network. EDS semi-quantitative analysis (Table 1) revealed that Ti is mainly composed of titanium, aluminum, and vanadium, while NT and PL are mainly composed of zirconium, yttrium, and oxygen. Aluminum was detected in AB and ABPL. GE and GEPL showed high silicon contents. HNP treatment consistently reduced surface carbon across groups.

**Figure 3:**
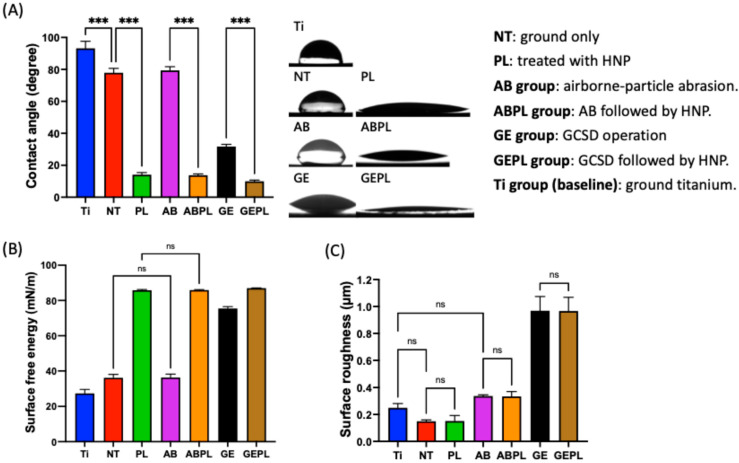
Surface characterization. (**A**) Static contact angle in distilled water. (**B**) SFE calculated using water and diiodomethane. (**C**) Ra. Symbols: *** *p* < 0.001; ns, not significant (*p* > 0.05).

Figure 3A,B summarize CA and SFE. Without HNP, Ti showed the highest water CA (93.16°), indicating the poorest hydrophilicity; NT and AB were intermediate, whereas GE exhibited the lowest CA (31.69°), demonstrating that the glass–ceramic coating significantly enhanced wettability (*p* < 0.05). After HNP (PL, ABPL, GEPL), CAs decreased significantly in all groups (*p* < 0.05). Nearly complete droplet spreading was observed on GEPL, which yielded the lowest CA (9.96°); PL and ABPL also approached 10°, consistent with a superhydrophilic surface. In addition, SFE was highest for GE among non-HNP groups (75.47 mN/m), significantly exceeding Ti, NT, and AB (*p* < 0.05). HNP further increased SFE in all groups, with GEPL reaching the highest value (86.98 mN/m), mirroring its minimal CA. Figure 3C shows Ra. NT had the lowest mean Ra (0.15 µm), with no significant difference from Ti (0.18 µm) or PL (0.18 µm) (*p* = 0.06). AB and ABPL increased to approx. 0.65 µm, while GE and GEPL exceeded 0.92 µm, indicating pronounced roughening from GCSD. Within each surface treatment, HNP did not alter Ra (*p* > 0.99).

### 3.2. Cell Attachment and Spreading

**Figure 4:**
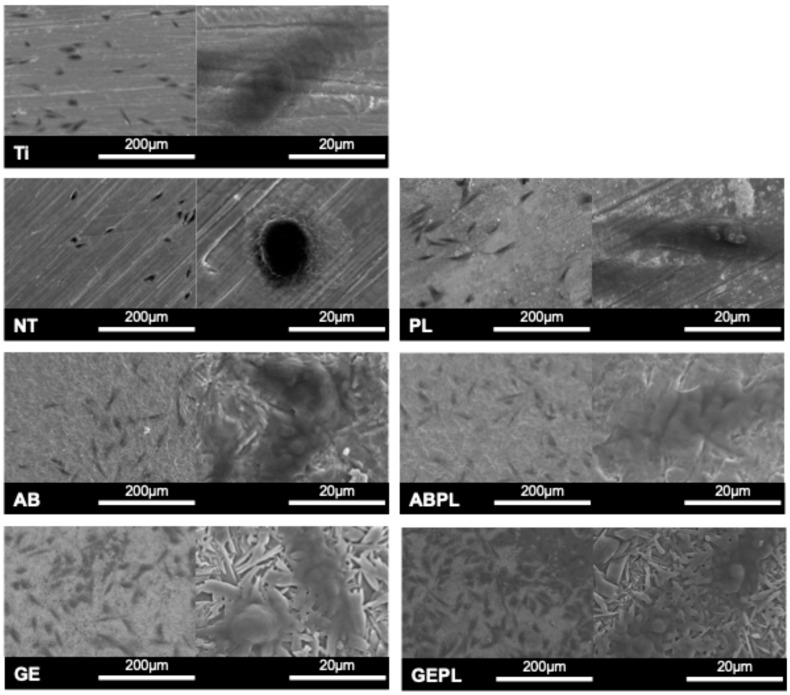
MG-63 attachment and morphology after 8 h. SEM at 200× (**left**) and 2000× (**right**).

Figure 4 shows MG-63 morphology after 8 h and Table 2 summarized the quantification of cell attachment area. Ti, NT, and PL showed low, sparse coverage (3.25%, 2.32%, and 7.88%), with predominantly round or mildly spread cells. AB and GE showed increased attachment (6.94% and 19.35%) with spindle-like morphology. HNP-combined treatments further enhanced attachment that ABPL reached 10.63%, and GEPL achieved 32.76% with uniformly distributed, highly elongated cells.

**Figure 5:**
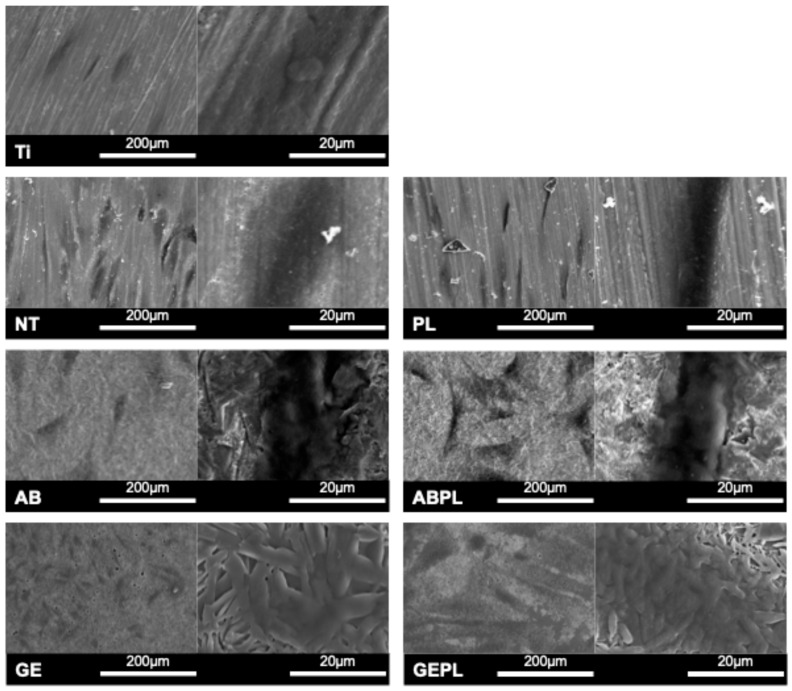
HGF-1 attachment and morphology after 24 h. SEM at 200× (**left**) and 2000× (**right**).

Figure 5 shows HGF-1 after 24 h and Table 2 summarizes the quantification of cell attachment area. Ti had the lowest, sparsest coverage (8.78%) with limited spreading. NT and PL showed more uniform distributions (19.31% and 22.19%) with spindle-like morphology aligned with polishing striations; spreading was more pronounced on PL. AB showed limited attachment (11.01%) and poor spreading, whereas GE reached 19.12% with higher counts but predominantly round/elliptical morphology. HNP-combined treatments improved attachment that ABPL increased to 14.70% with evident onset of spreading, while GEPL achieved 23.70% with markedly elongated cells.

### 3.3. Cellular Responses

**Figure 6:**
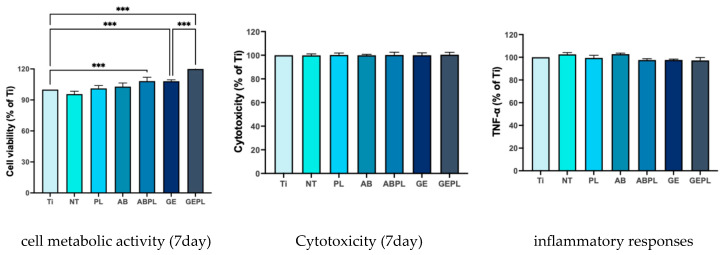
MG-63 responses on different surfaces. Data normalized to Ti on day 1. *** *p* < 0.001.

**Figure A1:**
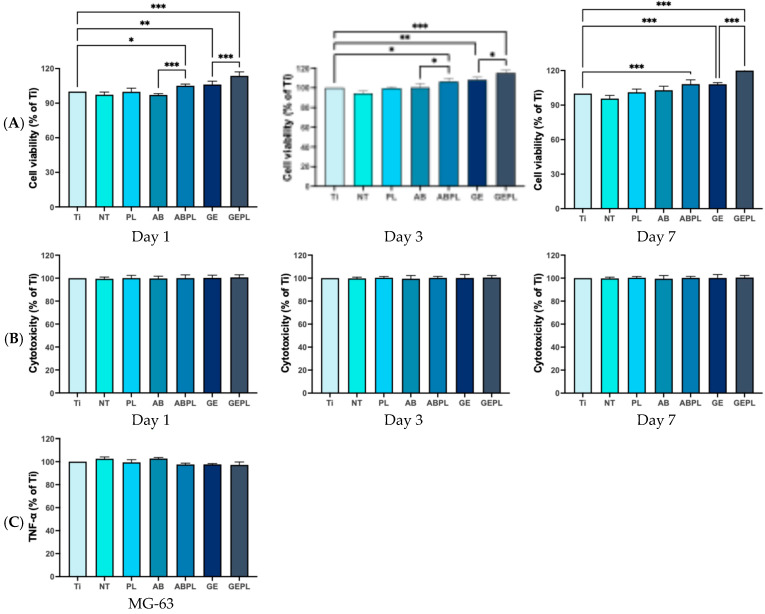
MG-63 responses on different surfaces. (**A**) Metabolic activity/viability. (**B**) Cytotoxicity. (**C**) TNF-α. Data normalized to Ti on day 1. * *p* < 0.05, ** *p* < 0.01, *** *p* < 0.001.

Figure 6 and Figure A1 show MG-63 viability. Using Ti as baseline, ABPL, GE, and GEPL were significantly higher on day 1 (*p* < 0.05), with HNP further increasing activity within the same substrates (AB vs. ABPL and GE vs. GEPL; *p* < 0.001), a pattern persisting through day 3. By day 7, ABPL, GE, and GEPL remained significantly higher than baseline (*p* < 0.001), whereas AB did not differ from ABPL (*p* = 0.06). It also shows cytotoxicity toward MG-63 that no significant differences were detected at any time point (*p* > 0.05). Moreover, the results show TNF-α, which remained comparable across groups (*p* > 0.05).

**Figure 7:**
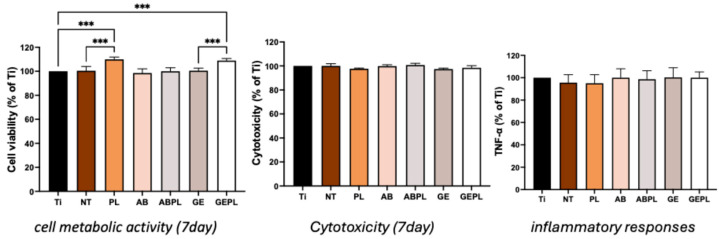
HGF-1 responses on different surfaces. Data normalized to Ti on day 1. *** *p* < 0.001.

**Figure A2:**
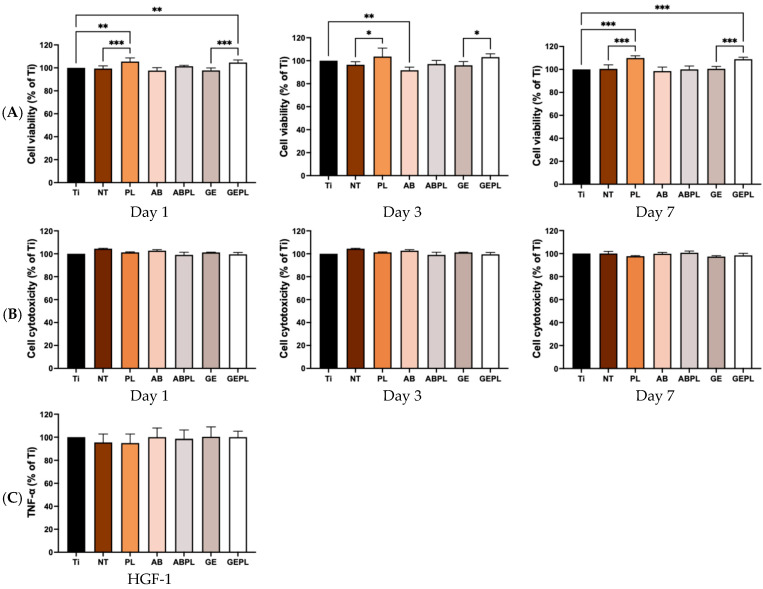
HGF-1 responses on different surfaces. (**A**) Metabolic activity/viability. (**B**) Cytotoxicity. (**C**) TNF-α. Data normalized to Ti on day 1. * *p* < 0.05, ** *p* < 0.01, *** *p* < 0.001.

Figure 7 and Figure A2 show HGF-1 viability at 1, 3, and 7 days. On day 1, most groups did not differ from baseline (*p* > 0.05) except PL and GEPL, which were significantly higher (*p* < 0.01) and exceeded their non-HNP counterparts (NT and GE; *p* < 0.05). On day 3, no group surpassed baseline (*p* > 0.05), although PL and GEPL remained higher than NT and GE (*p* < 0.05). By day 7, PL and GEPL were significantly higher than baseline (*p* < 0.001) and higher than their non-HNP counterparts (*p* < 0.001). It also shows cytotoxicity where all groups were comparable to baseline (*p* > 0.05), and the TNF-α where there were no increases relative to baseline (*p* > 0.05).

## 4. Discussion

GCSD uniformly deposits nanoscale lithium disilicate precursors on zirconia that, after sintering, form a dense, adherent glass–ceramic coating. Prior studies report a mean coating thickness of approximately 10 µm across sites, with good thickness uniformity and intimate contact confirmed by cross-sectional microscopy and elemental mapping, indicating a continuous silica-rich phase intimately bonded to the zirconia substrate [19]. In line with dental practice, GCSD is applied to the entire external surface of the implant abutment, providing uniform coverage of extra-gingival and transmucosal regions while the internal connection is masked to avoid any alteration in fit; this whole-surface approach supports consistent chairside handling and predictable cementation outcomes across all exposed areas. Liang et al. [20] pointed out that GCSD does not affect its suitability and there is no need to increase the setting value during customized production, suggesting that routine digital workflows can be retained without compensatory offsets or refitting. HF etching dissolves the siliceous glass matrix (Si–O bonds), revealing interwoven lithium metasilicate and lithium disilicate crystallites (Figure 2) and concurrently increasing surface roughness and reactivity [34]; meanwhile, the liberation of a silica-containing, micro-/nano-porous surface enhances silane coupling and promotes micromechanical interlocking with resin cement. Peng et al. [19] indicated that 5% HF for 100 s optimizes surface characteristics; given the abutment-oriented context requiring both biocompatibility and cell adhesion, this study adopted the same condition [22], as it balances crystal exposure with controlled material removal, preserves the integrity of the thin coating, and yields a clean, highly wettable surface compatible with subsequent silanization and resin bonding without necessitating additional airborne-particle abrasion or aggressive mechanical pretreatment.

Plasma primarily induces chemical activation by removing organic contaminants and introducing hydrophilic functionalities (e.g., –OH) or a thin oxide layer, with minimal impact on topography [29]. Owing to its compact form factor, operational flexibility, and lack of thermal or vacuum requirements, HNP is attractive for clinical and laboratory use [31,35]. Consistent with Huang et al. [31] showing that a 30 s HNP exposure markedly increases wettability and SFE of polymeric materials, this study applied the same condition across groups [29]. Quantitative EDS further confirmed efficient removal of surface organics after HNP (Figure 2).

FE-SEM revealed polished features on Ti, NT, and PL, whereas AB and ABPL showed irregular, markedly roughened microtopographies (Figure 2). GE and GEPL exhibited the canonical interlaced rod-/needle-like lithium silicate/disilicate texture, indicating robust coating adhesion and crystallization, in agreement with prior literature [34]. Given the abundance of polar Si–O–Si motifs in the glass–ceramic, hydrogen bonding with water is favored [34,36]; HF also removes residual contaminants, jointly enhancing hydrophilicity [19]. The CA data (Figure 3) showed hydrophobic Ti (CA > 90°), hydrophilic NT and AB (CA < 90°), and highly hydrophilic GE (CA < 30°), confirming a pronounced wettability gain with GCSD. Although HNP did not appreciably alter FE-SEM features or roughness (Figure 3), it produced pronounced chemical effects, such as removal of organics and generation of oxide/–OH functionalities, which increased SFE and wettability [15,29]. Post-HNP, CA values approached 10° across groups, with GEPL falling below 10°, indicative of a superhydrophilic surface that favors early cell attachment [31]. Overall, GCSD combined with HNP (GEPL) concurrently delivers micro-roughness and chemical activation.

Surface attributes (roughness, wettability, chemistry) synergistically regulate early attachment, spreading, proliferation, and differentiation [37,38]. MG-63 exhibited high density and well-spread morphologies on GEPL (Figure 4); ABPL showed similar trends to a lesser extent, aligning with evidence that rough and highly hydrophilic surfaces favor MG-63 early attachment [39]. HGF-1 displayed higher density and anisotropic spreading along polishing striations on NT and PL (Figure 5), consistent with reports that smooth surfaces with Ra < 0.2 µm and regular micro-grooves promote fibroblast guidance and metabolic activation [40,41]. Despite Ra > 0.2 µm, GEPL’s extreme hydrophilicity and crystalline microtexture supported HGF-1 attachment and spreading comparable to NT and PL [42,43].

Viability and cytotoxicity assays (Figure 6 and Figure 7) showed increased MG-63 metabolic activity on ABPL, GE, and GEPL, indicating that rough plus hydrophilic surfaces provide a stable growth niche; other groups resembled Ti baseline without toxicity. For HGF-1, PL and GEPL showed steady gains, likewise indicating a supportive microenvironment [42]. An indirect cytocompatibility assay was chosen to minimize substrate autofluorescence and protein adsorption artifacts. Direct, surface-specific effects are corroborated by cell attachment morphology and density analyses. Prior direct contact results on GCSD surfaces demonstrated non-toxicity [25]. Indirect assays revealed no differences in absorbance, suggesting no harmful leachables; TNF-α levels were comparable, supporting biosafety [13,14].

GCSD endows zirconia with a thin, approximately 10–12 µm lithium disilicate type coating, enabling the conventional glass–ceramic cementation pathway including HF etching, silanization, and resin cement, rather than reliance on airborne-particle abrasion with 10-MDP alone. Mechanistically, GCSD produces a silica-containing surface with Li_2_Si_2_O_5_ crystallites and a zirconium silicate ZrSiO_4_ interfacial layer at the zirconia/coating junction; together, these features support micromechanical interlocking after HF etching and robust silane coupling to resin matrices [21]. In vitro protocols typically use 5% HF for about 90–120 s to expose crystals and generate microporosity. After etching, a silane primer, e.g., methacrylate functional silanes, is applied, followed by luting with resin cements. Across multiple studies, GCSD followed by HF etch, silanization, and resin cementation yields bonding performance comparable to lithium disilicate [22,23]. Clinically, this confers a practical advantage: GCSD-modified zirconia can be cemented with the familiar glass–ceramic workflow, reducing technique variability and broadening cement selection [19,44]. The literature further highlights key handling points: first, etching time is critical for optimizing surface properties and consequently bonding on ceramic-coated zirconia; second, silanization is required to maximize chemical coupling with the exposed silica network; and third, artificial aging challenges confirm bond durability comparable to conventional glass–ceramics. Beyond adhesion metrics, fracture/debonding analyses in related zirconia veneer models show that surface-treatment-dependent cementation protocols are crucial for clinical stability, underscoring the value of GCSD in enabling a predictable HF to silane pathway rather than niche zirconia only chemistries. Collectively, these data position GCSD not as a mere surface add on but as an integration strategy that aligns high translucency zirconia with standard glass–ceramic cementation, thereby simplifying chairside procedures and improving interfacial reliability over time [19,21,22,23,44].

In summary, GCSD confers high hydrophilicity and tailored roughness via crystal exposure and a polar Si–O–Si network, while HNP chemically activates the surface, driving superhydrophilicity and decontamination. This combination (GEPL) co-optimizes microtopography and surface energy, enhancing MG-63/HGF-1 attachment and viability. Some limitations of this study are that we did not measure interleukin 6 (IL-6), a downstream cytokine that may be more characteristic for HGF-1 responses. Moreover, there is still a gap before the surface treatment performed in this experiment can be applied in dental practice, which requires more experiments to verify in the future. In future studies, we will include IL-6 and/or IL-8 under standardized stimulatory conditions and perform time-course analyses to provide a more comprehensive profile of gingival fibroblast inflammatory signaling across the tested surfaces. In addition, in the future, we need to include hydrothermal aging to validate long-term coating stability, systematic evaluation of bacterial adhesion/biofilm formation to assess antimicrobial potential and infection risk, and to develop functional, patient-specific abutments that improve treatment precision and efficiency.

## 5. Conclusions

GCSD forms a dense, stable lithium-disilicate-based phase on zirconia. Subsequent HF etching exposes crystals and generates a microtopography with high roughness and reactivity, indicating robust coating–substrate integration. HNP does not alter topography but markedly increases wettability and introduces oxygen-containing functionalities, thereby strengthening cell–material interfacial interactions. Biologically, GCSD combined with HNP (GEPL) enhanced MG-63 attachment and spreading and HGF-1 cells also adhered and grew stably without cytotoxicity or inflammatory responses. Based on the results of this in vitro study, GEPL improves zirconia surface physicochemical properties and bone metabolic activity while preserving mechanical strength and processability, supporting its potential as an option for implant abutments and adhesive restorations.

## Figures and Tables

**Table 1:** Surface element distribution percentage (%).

	C	O	Al	Si	Y	Zr	Ti	V	Total
Ti	6.48	0.00	5.85	0.00	0.00	0.00	85.06	2.60	100.00
NT	12.02	23.44	0.00	0.00	4.34	60.21	0.00	0.00	100.00
PL	4.76	24.59	0.00	0.00	4.60	66.05	0.00	0.00	100.00
AB	12.30	25.94	1.65	0.00	3.40	56.72	0.00	0.00	100.00
ABPL	8.08	28.20	2.11	0.00	3.43	58.18	0.00	0.00	100.00
GE	11.60	52.98	0.87	31.67	0.00	2.87	0.00	0.00	100.00
GEPL	6.67	53.35	1.29	34.36	0.00	4.34	0.00	0.00	100.00

**Table 2:** Quantification of cell attachment area (%) of MG-63 and HGF-1 cells.

	Ti	NT	PL	AB	ABPL	GE	GEPL
MG-63	3.25 ± 0.37	2.32 ± 0.53	7.88 ± 0.55	6.94 ± 1.11	10.63 ± 1.57	19.35 ± 1.37	32.76 ± 1.25
HGF-1	8.78 ± 0.58	19.31 ± 0.97	22.19 ± 1.39	11.01 ± 0.62	14.70 ± 1.07	19.12 ± 1.16	23.70 ± 0.82

## Data Availability

The original contributions presented in this study are included in the article. Further inquiries can be directed to the corresponding authors.
